# *APOE* ε4-*TOMM40* ‘523 haplotypes and the risk of Alzheimer’s disease in older Caucasian and African Americans

**DOI:** 10.1371/journal.pone.0180356

**Published:** 2017-07-03

**Authors:** Lei Yu, Michael W. Lutz, Robert S. Wilson, Daniel K. Burns, Allen D. Roses, Ann M. Saunders, Jingyun Yang, Chris Gaiteri, Philip L. De Jager, Lisa L. Barnes, David A. Bennett

**Affiliations:** 1 Rush Alzheimer's Disease Center, Rush University Medical Center, Chicago, Illinois, United States of America; 2 Department of Neurological Sciences, Rush University Medical Center, Chicago, Illinois, United States of America; 3 Department of Neurology, Duke University School of Medicine, Durham, North Carolina, United States of America; 4 Zinfandel Pharmaceuticals, Inc., Research Triangle Park, North Carolina, United States of America; 5 Center for Translational and Computational Neuroimmunology, Department of Neurology, Columbia University Medical Center, New York, New York, United States of America; 6 Program in Medical and Population Genetics, Broad Institute, Cambridge, Massachusetts, United States of America; Oslo Universitetssykehus, NORWAY

## Abstract

Patterns of linkage between the ε4 allele of Apolipoprotein E (*APOE*) and ‘523 poly-T alleles in the adjacent gene, *TOMM40*, differ between Caucasian and African Americans. The extent to which this difference affects the risk of Alzheimer’s disease (AD) is unclear. We compared the *APOE* ε4-*TOMM40* ‘523 haplotypes between older Caucasian and African Americans, and examined their relationship with AD dementia. Data came from three community based cohort studies of diverse participants. *APOE* genotypes were determined by polymorphisms of rs429358 and rs7412. *TOMM40* ‘523 genotypes were defined by the poly-T repeat length of rs10524523 (short [‘523-S]: poly-T ≤ 19, long [‘523-L]: 20 ≤ poly-T ≤ 29, and very long [‘523-VL]: poly-T ≥ 30). Cox proportional hazards models examined the effect of haplotype variation on the risk of incident AD dementia. A total of 1,848 Caucasian and 540 African American individuals were included in the study. In Caucasians, nearly none (0.8%) of the non-ε4 carriers and almost all (94.2%) of the ε4 carriers had ‘523-L. The classification was highly concordant. Each ε4 allele doubled the risk for AD dementia and the dose effect was evident. Almost identical effect size and effect pattern were observed for *TOMM40* ‘523-L. In African Americans, nearly none (1.1%) of the non-ε4 carriers had ‘523-L, but only 47.8% of the ε4 carriers had ‘523-L. The concordance was weaker compared with Caucasians. The effect patterns on incident AD dementia differed distinctively between ε4 and ‘523-L carriers. Further, both genotypic and allelic data support that among African Americans the ε4-‘523-L haplotype had stronger effect on risk of AD dementia than other ε4-‘523 haplotypes.

## Introduction

Apolipoprotein E (*APOE*) ε4 is by far the most replicated risk allele for late onset Alzheimer’s disease (AD). Compared with Caucasians, the allele frequency of ε4 is higher among African Americans but its effect on the disease susceptibility appears weaker[1–3]. This inconsistency may be attributable to race specific variation of haplotypes across *APOE* and neighboring genes. For instance, it has been reported that among Caribbean Hispanic ε4 carriers, subjects with the HpaI+ variant in the downstream *APOC1* gene are more likely to have AD, but the same association is not observed among African Americans[4]. Recent data suggest that the haplotypes between *APOE* and a poly-T variant in another adjacent gene, Translocase of Outer Mitochondria Membrane (*TOMM40*) ‘523, together provide better precision in estimating the age of AD onset[5]. The ‘523 variant is located in the intron of *TOMM40*. Depending on the poly-T repeat length, individual ‘523 alleles can be grouped into 3 classes: Short (‘523-S: Poly-T repeat ≤ 19), Long (‘523-L: 20 ≤ poly-T repeat ≤ 29), and Very Long (‘523-VL: poly-T repeat ≥ 30)[6]. Genetic studies show that varying ‘523 poly-T repeat length differentiates AD susceptibility among Caucasian ε3/ε3 carriers[7, 8]. Notably, the *APOE* and *TOMM40* ‘523 haplotypes differ between Caucasian and African Americans[9]. In Caucasians, ε4 is almost perfectly linked with the ‘523-L allele. By contrast, in African Americans, ε4 is commonly linked to ‘523-S in addition to the ‘523-L allele. The extent to which such difference affects AD risk in African Americans has not been reported.

In this study, using data from a large sample of prospective longitudinal studies of aging, we compared the distributions of *TOMM40* ‘523 in linkage with *APOE* ε4 between older Caucasian and African Americans, and examined their relationship with incident AD dementia. Since *APOE* ε4 and *TOMM40* ‘523-L are in strong linkage disequilibrium (LD) among Caucasians, it is expected that the two alleles will demonstrate a similar effect on AD dementia in this population. By contrast, the heterogeneity of the ε4-‘523 haplotypes among African Americans allows us to investigate the role of the ε4-‘523-L haplotype in relation to AD susceptibility.

## Materials and methods

### Study participants

Participants came from three community-based longitudinal cohort studies of diverse participants, the Religious Orders Study (ROS)[10], the Rush Memory and Aging Project (MAP)[11], and the Minority Aging Research Study (MARS)[12]. The studies were approved by the Institutional Review Board of Rush University Medical Center. Participants were enrolled without known dementia and each participant signed an informed consent and agreed to annual clinical evaluations. The ROS and MAP participants were predominantly Caucasian Americans and the MARS participants were all African Americans. Importantly, all three studies are conducted by the same team of investigators and share a large common core of testing batteries and uniform structured clinical evaluations. This makes it possible for a combined analysis.

At the time of this study, 1,948 Caucasian participants free of AD dementia at baseline had completed at least 1 follow-up clinical evaluation. Genotyping data were available on 1,886 (96.8%) of the participants. Considering the conflicting effects between the ε2 and ε4 alleles on AD dementia, ε2/4 carriers (N = 38) were excluded. As the result, statistical analyses were conducted in the remaining 1,848 Caucasian participants. The mean age at baseline was 78.4 years (standard deviation [SD]: 7.4, range: 54.3–102.1). 71.2% were females and the mean years of education was 16.3 (SD: 3.5, range: 5–30). The average length of follow-up for the Caucasian participants was 8 years (SD = 5.1, range: 1–22).

Separately, genotyping data were available on 568 (98.8%) of the 575 African American participants who were dementia free at baseline and had completed at least 1 follow-up evaluation. We excluded 28 ε2/4 carriers and focused the analyses on the remaining 540 African Americans. For the African American participants, the mean baseline age was 73.4 years (SD: 6.6, range: 58.9–97.6). 77.6% were females and the mean education was 14.8 years (SD: 3.4, range: 0–30). The average length of follow-up was 5.9 years (SD = 3.6, range: 1–16).

### *APOE* ε4 and *TOMM40* ‘523 genotyping

DNA was extracted from peripheral blood mononuclear cells (PBMC) or post-mortem brain tissue. Genotyping was performed by Polymorphic DNA Technologies (Alameda, California), blinded to all clinical data. The *APOE* genotypes were determined by the two polymorphisms of rs429358 (codon 112) and rs7412 (codon 158) at exon 4 of the *APOE* gene. The *TOMM40* ‘523 genotypes were determined by the polymorphism rs10524523 at intron 6 of the *TOMM40* gene (chr19:44,899,792–44,899,826, human genome reference assembly GRCh38/hg38). Based on the the poly-T repeat length, each ‘523 allele was categorized into Short (‘523-S), Long (‘523-L) and Very Long (“523-VL), as previously defined[6]. In this study, we primarily focused on the effect of ε4 in linkage with *TOMM40* ‘523.

Distinct from the exclusive linkage between ε4 and ‘523-L in Caucasian Americans, other ‘523 alleles, ‘523-S in particular, have been reported in linkage with ε4 among African Americans. We therefore obtained strand specific (phased) haplotype data in a subset of African American ε3/4 heterozygotes in an effort to further explore how the strand specific ε4-‘523 haplotype variations differ in relation to AD dementia.

### Clinical diagnosis of AD dementia

Participants underwent a detailed cognitive testing and a uniform structured clinical evaluation each year. Cognitive assessment results were reviewed by a neuropsychologist for signs of impairment in various domains. Participants were examined by a clinician. After reviewing all available data, an annual diagnosis was made by the clinician. Diagnosis of AD follows the recommendation of the joint working group of the National Institute of Neurological and Communicative Disorders and Stroke and the Alzheimer's Disease and Related Disorders Association[13] and the decision rules for implementing these criteria have been described previously[14]. Briefly, the AD diagnosis requires a history of cognitive decline and evidence of impairment in multiple cognitive domains including memory.

### Statistical analysis

Student *t* and Chi-square tests described demographic and genetic differences between Caucasian and African Americans. Primary analyses were stratified by race. Cohen's κ coefficients[15] evaluated the concordance between *APOE* ε4 and *TOMM40* ‘523-L genotypes. Cox proportional hazards models examined the associations of genetic variants with incident AD dementia. In these models, the outcome variable was time in years to incident AD dementia (event time). Time was right-censored at death or last clinical evaluation for participants who were never diagnosed with AD dementia. We first examined the ε4 effect by fitting a model including terms for ε3/4 heterozygous and ε4/4 homozygous. The dose effect was assessed by estimating the difference between the effect of ε4/4 and twice the effect of ε3/4. Next, we repeated the model for ‘523-L in lieu of ε4. Both models were adjusted for baseline age, sex, and education. Finally, by leveraging phased haplotype data from 83 African American ε3/4 heterozygotes, we applied Cox proportional hazards model to evaluate the associations of strand specific ε4-‘523 haplotype variations with incident AD dementia.

All the analyses were conducted using SAS/STAT programs (SAS Institute, Cary, NC). Statistical significance was determined *a priori* at the nominal level of α = 0.05.

## Results

### Characteristics of study participants

Characteristics of study participants were summarized in Table 1. Compared with African Americans, Caucasian Americans in this study were older and had more years of education (both *p*s <0.001). Percent female participants were higher among African Americans (*p* = 0.003). On average, Caucasian Americans were followed 2 years longer than African Americans (*p*<0.001). During the follow-ups, 27.8% of Caucasian Americans and 10.4% of African Americans were diagnosed with AD dementia.

**Table 1:** Characteristics of the study participants.

	Caucasian Americans	African Americans
N	1,848	540
Baseline age (yr)	78.4 (7.4)	73.4 (6.6)
Females	1,316 (71.2%)	419 (77.6%)
Education (yr)	16.3 (3.5)	14.8 (3.4)
Length of follow-up (yr)	8.0 (5.1)	5.9 (3.6)
Incident AD dementia	513 (27.8%)	56 (10.4%)
*APOE*		
ε2/2	10 (0.5%)	5 (0.9%)
ε2/3	251 (13.6%)	79 (14.6%)
ε3/3	1170 (63.3%)	278 (51.5%)
ε3/4	390 (21.1%)	149 (27.6%)
ε4/4	27 (1.5%)	29 (5.4%)
*TOMM40*		
‘523 S/S	367 (19.9%)	237 (43.9%)
‘523 S/L	188 (10.2%)	59 (10.9%)
‘523 S/VL	691 (37.4%)	164 (30.4%)
‘523 L/L	30 (1.6%)	7 (1.3%)
‘523 L/VL	187 (10.1%)	23 (4.3%)
‘523 VL/VL	385 (20.8%)	50 (9.3%)

Means and standard deviations (SD) or N (%)

The ε4 allele was present in 22.6% of the Caucasian Americans, of which 21.1% were ε3/4 heterozygous and 1.5% were ε4/4 homozygous. The presence of ε4 was higher among the African Americans (33.0%, *p*<0.001), of which 27.6% were ε3/4 heterozygous and 5.4% were ε4/4 homozygous. The ‘523-L allele was present in 21.9% of the Caucasian Americans, comparable to the percentage of ε4. Of the ‘523-L carriers, approximately half (10.2%) were ‘523 S/L, another half were L/VL (10.1%), and only a small number of ‘523-L carriers were L/L homozygous (1.6%). The presence of the ‘523 L allele was lower among the African Americans (16.5%, *p* = 0.006), of which a majority were of S/L genotype (10.9%), followed by L/VL (4.3%) and L/L (1.3%).

### *APOE* ε4 and *TOMM40* ‘523-L with AD dementia in Caucasian Americans

Consistent with the linkage between ε4 and ‘523-L, nearly none (0.8%) of the Caucasian non-ε4 carriers had ‘523-L, whereas 94.2% of the Caucasian ε4 carriers had ‘523-L (Table 2). The classification was highly concordant (Cohen’s κ = 0.94, 95% Confidence Interval [CI] = 0.93–0.96). Compared with Caucasian non-ε4 carriers, the ε3/4 heterozygotes had almost doubled risk for incident AD dementia (Hazard Ratio [HR] = 1.85, 95% CI = 1.51–2.26, *p*<0.001) and the ε4/4 homozygotes had quadrupled risk (HR = 4.04, 95% CI = 2.19–7.46, *p*<0.001) (Table 3). The difference between the estimated effect size of ε4/4 and twice the estimated effect size of ε3/4 was not significant (*p* = 0.632), supporting a dose effect of ε4 on AD susceptibility.

**Table 2:** Distribution of *TOMM40* ‘523 genotypes by *APOE* ε4*.

*TOMM40* ‘523	Caucasian Americans				African Americans			
	Non ε4 carriers		ε4 carriers		Non ε4 carriers		ε4 carriers	
	Frequency	Percent	Frequency	Percent	Frequency	Percent	Frequency	Percent
S/S	365	25.5	2	0.5	194	53.6	43	24.2
S/L	6	0.4	182	43.7	4	1.1	55	30.9
S/VL	682	47.7	9	2.2	130	35.9	34	19.1
L/L	0	0	30	7.2	0	0	7	3.9
L/VL	6	0.4	181	43.4	0	0	23	12.9
VL/VL	372	26.0	13	3.1	34	9.4	16	9.0
Total	1,431	100	417	100	362	100	178	100

* *APOE* ε2/4 not included.

**Table 3:** *APOE* ε4 and *TOMM40* ‘523-L with AD dementia in Caucasian Americans.

	HR (95% CI, *p*)	HR (95% CI, *p*)
Age	1.13 (1.11–1.14, <0.001)	1.13 (1.11–1.14, <0.001)
Male sex	0.97 (0.78–1.19, 0.738)	0.95 (0.77–1.17, 0.629)
Education	1.01 (0.99–1.04, 0.340)	1.01 (0.99–1.04, 0.319)
ε3/4 heterozygosity	1.85 (1.51–2.26, <0.001)	-
ε4/4 homozygosity	4.04 (2.19–7.46, <0.001)	-
‘523-L heterozygosity	-	1.89 (1.54–2.32, <0.001)
‘523-L homozygosity	-	3.80 (2.14–6.73, <0.001)

HR: Hazard ratio; CI: Confidence interval

The result in column 2 was from a Cox proportional hazards model which examined *APOE* ε4 genotypes on incident AD dementia, and the result in column 3 was from a separate Cox model which examined *TOMM40* ‘523-L genotypes on incident AD dementia.

**Fig 1:**
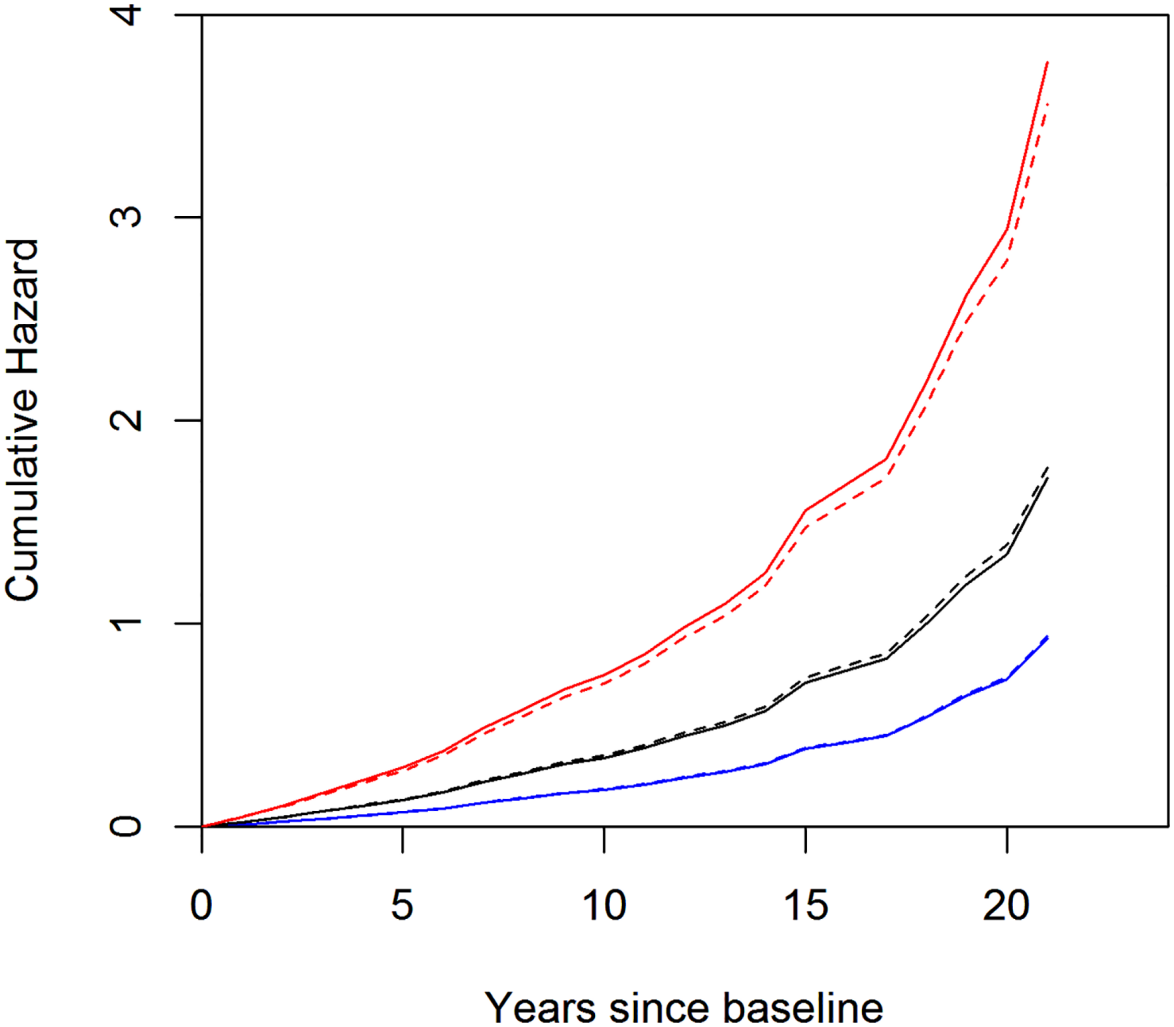
Cumulative hazards of incident AD dementia by *APOE* ε4 and *TOMM40* ‘523-L genotypes in Caucasian Americans. The figure illustrates cumulative hazards of incident AD dementia for representative female Caucasians with mean age and education. The blue solid curve represents cumulative hazard for non-ε4 carriers, the black solid curve for ε4 heterozygotes, and the red solid curve for ε4 homozygotes. Superimposed are the blue dash curve representing cumulative hazards for non-‘523-L carriers, the black dash curve for ‘523-L heterozygotes, and the red dash curve for ‘523-L homozygotes.

Almost identical effect size and effect pattern were observed for *TOMM40* ‘523-L in Caucasian Americans (Fig 1). The hazard ratios for the 523-L heterozygotes and homozygotes were 1.89 and 3.80 respectively (both *p*s<0.001). The dose effect of the ‘523-L allele was also evident, and the estimated effect of the 523-L homozygosity was equivalent to twice the effect of the 523-L heterozygosity (*p* = 0.859).

### *APOE* ε4 and *TOMM40* ‘523-L with AD dementia in African Americans

The linkage between ε4 and ‘523-L was much weaker in African Americans, with the two variants being less concordant (Cohen’s κ = 0.53, 95% CI = 0.46–0.61). Specifically, while 1.1% of the non-ε4 carriers had ‘523-L, only 47.8% of the ε4 carriers had ‘523-L (Table 2).

**Fig 2:**
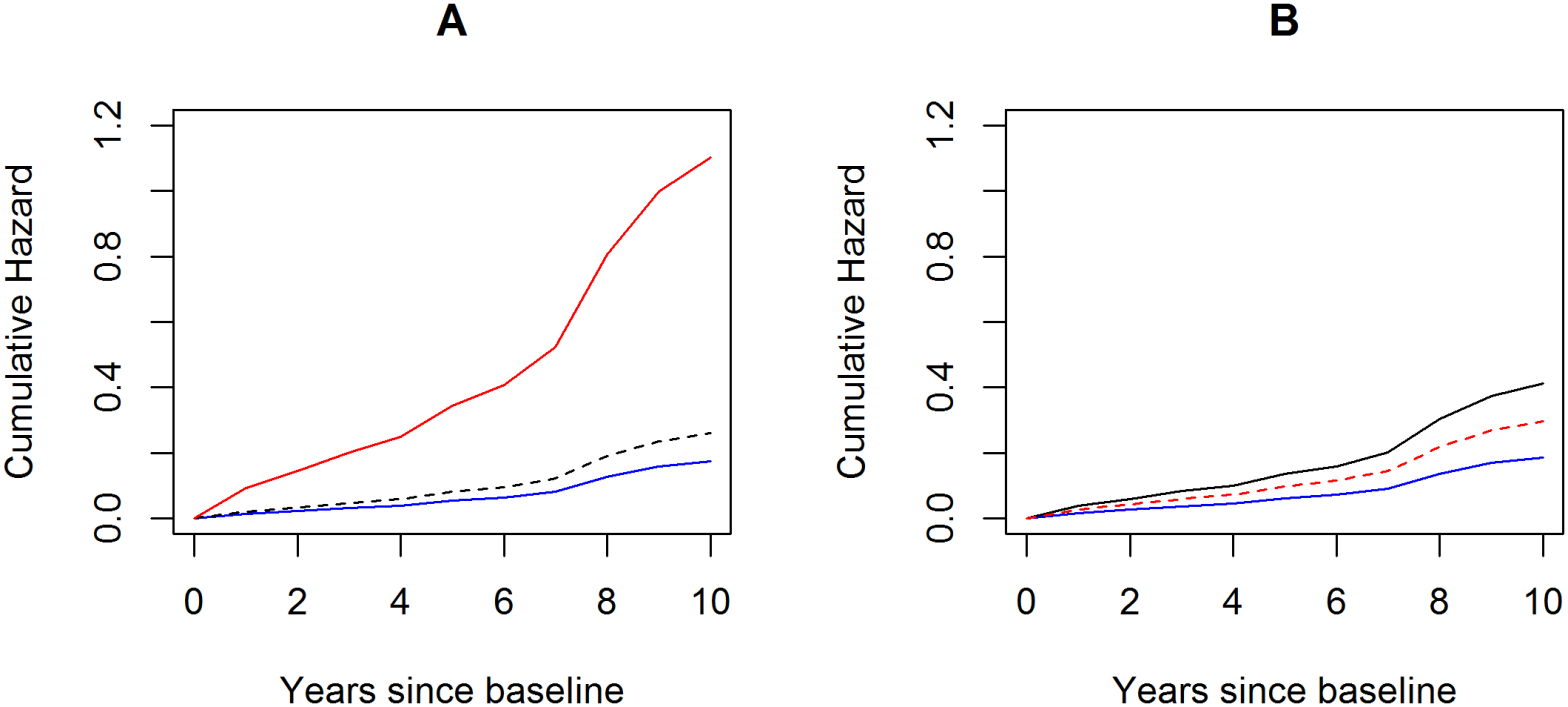
Cumulative hazards of incident AD dementia by *APOE* ε4 and *TOMM40* ‘523-L genotypes in African Americans. The figure illustrates cumulative hazards of incident AD dementia for representative female African Americans with mean age and education. For panel A, the blue solid curve represents cumulative hazard for non-ε4 carriers, the black dash curve for ε4 heterozygotes, and the red solid curve for ε4 homozygotes. For panel B, the blue solid curve represents cumulative hazards for non-‘523-L carriers, the black solid curve for ‘523-L heterozygotes, and the red dash curve for ‘523-L homozygotes.

The Cox proportional hazards model shows that while the ε3/4 heterozygotes among African Americans had higher risk for AD dementia, the estimated hazard ratio was not significant (*p* = 0.195) (Table 4). By contrast, the ε4/4 homozygotes increased the AD risk by over six fold (HR = 6.32, 95% CI = 2.76–14.45, *p*<0.001). The lack of dosage effect for *APOE* ε4 in African Americans may be due to small sample size, and the test that assessed the difference between the effect of ε4/4 and twice the effect of ε3/4 was inconclusive. Notably, The effect pattern of *TOMM40* ‘523-L on AD dementia differed distinctively from that of *APOE* ε4 (Fig 2). The ‘523-L heterozygosity doubled the risk for incident AD dementia (HR = 2.21, 95% CI = 1.20–4.08, *p* = 0.011), but we did not detect a significant association for ‘523-L homozygosity (*p* = 0.650).

**Table 4:** *APOE* ε4 and *TOMM40* ‘523-L with AD dementia in African Americans.

	HR (95% CI, *p*)	HR (95% CI, *p*)
Age	1.11 (1.06–1.15, <0.001)	1.10 (1.06–1.14, <0.001)
Male sex	1.48 (0.83–2.66, 0.186)	1.49 (0.83–2.66, 0.183)
Education	1.01 (0.93–1.10, 0.789)	1.02 (0.94–1.10, 0.703)
ε3/4 heterozygosity	1.49 (0.82–2.73, 0.195)	-
ε4/4 homozygosity	6.32 (2.76–14.45, <0.001)	-
‘523-L heterozygosity	-	2.21 (1.20–4.08, 0.011)
‘523-L homozygosity	-	1.60 (0.21–12.0, 0.650)

HR: Hazard ratio; CI: Confidence interval

The result in column 2 was from a Cox proportional hazards model which examined *APOE* ε4 genotypes on incident AD dementia, and the result in column 3 was from a separate Cox model which examined *TOMM40* ‘523-L genotypes on incident AD dementia.

Since almost all of the non-ε4 African Americans did not carry ‘523-L allele and approximately half of the ε4 carriers had ‘523-L allele, we further split the ε4 carriers into those with and without the ‘523-L allele. 8.4% of the 358 African American non-ε4 carriers were diagnosed with AD dementia, and this percentage increased to 10.8% and 17.7% respectively for the ε4 carriers without (N = 93) and with the ‘523-L allele (N = 85). Results from the Cox proportional hazards model show that, compared with the non- ε4 carriers, African Americans ε4 carriers with the ‘523-L alleles had over twice the risk for incident AD dementia (HR = 2.34, 95% CI = 1.24–4.42, *p* = 0.009). The risk for the ε4 carriers in absence of ‘523-L allele was weaker and not significant (HR = 1.66, 95% CI = 0.80–3.44, *p* = 0.177).

To assess the robustness of our findings considering differences between Caucasian and African Americans in this study, we performed secondary analyses using a subset of individuals that were 1-to-1 matched by age, sex, education, length of follow-up as well as vital status. A total of 464 Caucasian and 464 African Americans were included. Notably, while statistical difference was no longer observed in any of the matching variables (all *p*s>0.05), results were consistent with those using the full dataset. In Caucasians, there was a high concordance between ε4 and ‘523-L (Cohen’s κ = 0.94). Nearly none (0.6%) of the non-ε4 carriers and almost all (92.4%) of the ε4 carriers had ‘523-L. The effect size and effect pattern on incident AD dementia were similar between ε4 and ‘523-L (Table A in S1 File). In African Americans, we observed a weak concordance between ε4 and ‘523-L (Cohen’s κ = 0.55). Nearly none (1.3%) of the non-ε4 carriers had ‘523-L, but only 49% of the ε4 carriers had ‘523-L. The results in relation to incident AD dementia for African Americans were essentially the same as reported in the full dataset, such that the effect patterns on incident AD dementia differed between ε4 and ‘523-L carriers (Table B in S1 File). The ε3/4 heterozygosity had higher risk for AD dementia, but was not significant. By contrast, the ε4/4 homozygosity increased the AD risk by over 7 fold, and was highly significant. The ‘523-L heterozygosity doubled the risk for incident AD dementia, but we did not detect a significant association for ‘523-L homozygosity. Further, ε4 coupled with ‘523-L had a stronger effect on the risk of AD dementia than ε4 in the absence of ‘523-L.

#### Strand specific ε4 and ‘523-L linkage patterns in African Americans

In an exploratory analysis, we examined phased ε4-‘523 haplotype variations in 83 African American ε3/4 heterozygotes (Table 5). In this sample, a majority (66.3%) of the ε4 alleles were linked to the ‘523-L allele, 24.1% linked to ‘523-S, and 9.6% linked to ‘523-VL. Similar to the genotypic results, we observed that compared with non-ε4 carriers, African Americans with strand specific ε4-‘523-L haplotype show stronger effect on the increased risk for AD dementia (HR = 2.01, 95% CI = 0.96–4.20, *p* = 0.064) than those with either ε4-‘523-S or ε4-‘523-VL haplotypes (HR = 1.27, 95% CI = 0.38–4.27, *p* = 0.696).

**Table 5:** Strand specific haplotypes for ε3/4 heterozygotes in African Americans.

Group	Haplotype (allele 1)	Haplotype (allele 2)	Frequency	Percent
1: ε4_L	ε3_L	ε4_L	2	2.4
1: ε4_L	ε3_S	ε4_L	36	43.4
1: ε4_L	ε3_VL	ε4_L	1	1.2
1: ε4_L	ε4_L	ε3_L	1	1.2
1: ε4_L	ε4_L	ε3_S	1	1.2
1: ε4_L	ε4_L	ε3_VL	14	16.9
2: ε4_VL	ε3_L	ε4_VL	1	1.2
2: ε4_VL	ε3_S	ε4_VL	6	7.2
2: ε4_VL	ε3_VL	ε4_VL	1	1.2
3: ε4_S	ε3_S	ε4_S	3	3.6
3: ε4_S	ε4_S	ε3_L	1	1.2
3: ε4_S	ε4_S	ε3_S	1	1.2
3: ε4_S	ε4_S	ε3_VL	15	18.1
Total			83	100

## Discussion

In this study, we examined the haplotypes between *APOE* ε4 and the neighboring *TOMM40* ‘523 variant in a large sample of community based older Caucasian and African Americans, and we interrogated their associations with incident AD dementia. Several observations were made.

We confirmed the linkage between ε4 and ‘523-L in Caucasians Americans. This linkage is highly concordant, such that almost all the ε4 carriers had presence of the ‘523-L allele and almost all the non-ε4 carriers were absent of the ‘523-L allele. When modeled for the associations with incident AD dementia, ε4 and ‘523-L share very similar effect size and effect pattern as expected. In both cases, every single allele doubles the risk for AD dementia and the dose effect is evident. The two ‘523-L heterozygosities (‘523 S/L and ‘523 L/VL) show the same strength in association with incident AD dementia. This strong linkage between ε4 and ‘523-L among Caucasian Americans probably accounts for the lack of association of the poly-T repeat length with age of AD onset in a previous report[16]. Some evidence suggests that the three *TOMM40* ‘523 genotypes (S/S, S/VL and VL/VL) that are exclusive to the non- ε4 carriers may further differentiate age of onset for late onset AD[17], but the results have not been conclusive[7, 18].

It is noted that the effect size of *APOE* ε4 on the risk of AD dementia in Caucasians can differ across studies due to a variety of reasons ranging from population or sample differences to phenotypic variation. A previous study reported an odds ratio of 2.7 for ε3/4 and 12.5 for ε4/4, relative to ε3/3[2]. The study differs from ours in several important ways. First, the prior study is a meta-analysis that combined data from 40 participating centers with different study designs. As the result, the samples are more heterogeneous. Indeed, the authors reported that study specific odds ratios of AD for ε4 carriers versus non-carriers varied, and the associations were weaker for population/community based studies. Second, the prior study was based on cross-sectional data where prevalent AD cases are mixed with incident cases. By contrast, our data came from community based cohorts and we focused on incident AD. All individuals included in the analyses were free of dementia at baseline and followed longitudinally. The effect size of ε4 reported in our study is in general consistent with those from similar population or community based studies on incident AD [19–21]. Finally, our subjects are older and the effect of ε4 tends to attenuate in old age.

We observed that the frequencies of *TOMM40* ‘523 genotypes differ distinctively between African and Caucasian Americans. There was an enrichment of '523-S allele in African Americans. In particular, the percent '523-S/S carriers in African Americans was more than doubled compared with Caucasian Americans. This result is consistent with an earlier report that the ‘523-S allele is more prevalent in African Americans than whites or Hispanics[22].

We also observed that the strong linkage between ε4 and ‘523-L haplotype was absent in African Americans. While nearly none of the non-ε4 carriers had the ‘523-L allele, only less than half of the ε4 carriers had ‘523-L. Consequently we could examine the effect patterns of haplotype variations on incident AD dementia. In fact, data at both genotypic and allelic level suggest that ε4-‘523-L haplotype among African Americans has a stronger effect on the susceptibility of AD dementia than other ε4-‘523 haplotypes. This finding helps further elucidate the relationship between *APOE* ε4 and AD dementia in African Americans.

The reports on ε4 and AD in African Americans have been inconsistent[1, 23–26]. Multiple studies show that the ε4 effect on AD tends to be weaker in African Americans than Caucasian Americans[2, 27]. In addition, the strength of the ε4 allele is even weaker in African Yoruban than African Americans[28–30]. These findings raise the possibility that the ε4 effect in African Americans is likely affected by the degree of admixture with Caucasian or other populations. One hypothesis is that admixture leads to haplotype variations surrounding the *APOE* locus. The *cis* ε4 haplotype difference due to population stratification is not new. One earlier study reported an *APOE* ε4 and *APOC1* HpaI+ haplotype that differs in frequency between ADs and controls, and such difference is only observed in Caribbean Hispanics, but not in African Americans[6]. Variation in *TOMM40* ‘523—*APOE* haplotypes among African Americans may well be just another example. Indeed, while the ε4-*TOMM40* ‘523-L haplotype predominates in Caucasians, two additional haplotypes have been reported in Yorubans. The linkage between ε4 and multiple ‘523 alleles in African Americans represents a genetic crossover between Caucasian and Western African populations[9]. Data from this study support the theory that, of the three ε4- *TOMM40* ‘523 haplotypes, the two originated from the Western African population are less potent than the ε4- *TOMM40* ‘523-L haplotype in affecting the susceptibility of AD. Since genetic risk factors in admixed populations including African Americans are highly dependent on ancestral background, future work is warranted to identify local ancestry in the *APOE*-*TOMM40* region and to investigate its implication in the risk of AD.

Extensive evidence suggests that the *APOE* gene is involved in amyloid deposition, a neuropathologic hallmark of AD, via amyloid-β clearance [31, 32]. The *APOE* ε2, ε3, and ε4 alleles encode three protein isoforms (apoE2, apoE3 and apoE4) which show differential response for amyloid-β kinetics. In mouse models, apoE4 increases amyloid-β deposition relative to apoE2 or apoE3[33, 34]. In humans, *APOE* ε4 carriers have more severe burden of amyloid plaques[35, 36], and the effect of ε4 on cognition and AD dementia is largely mediated by amyloid and downstream neurofibrillary tangles [37, 38]. The biochemical mechanism by which the *TOMM40* ‘523 variant affects AD pathophysiology is an area of active research[39]. Structural DNA variations, especially those in intronic or intergenic regions like *TOMM40* ‘523, most likely exert their effects by altering gene transcription efficiency, the timing of transcription, transcript stability, transcript splicing, or possibly by changing patterns of epigenomic modification[40–46]. There is support for the idea that short structural variants may be involved in human diseases [47–49]. It has been demonstrated that *TOMM40* ‘523 affects the expression levels of *APOE* and *TOMM40* mRNAs in the temporal and occipital cortexes of late onset AD patients and normal controls[50], and the effect of the *TOMM40* poly-T variation on transcription regulation was recapitulated in a cell-based luciferase reporting system[51]. Gene expression studies suggest that the number of protein import channels per mitochondrion may be regulated by *TOMM40* poly-T variants. Bekris *et al*. described a complex transcriptional regulatory region for *TOMM40* and *APOE* expression that extends throughout both genes and is influenced by multiple polymorphisms including the *TOMM40* poly-T locus[52]. It has been suggested that a relatively modest change in mRNA expression may produce pathology that accumulate over time and is expressed clinically in later age.

To our knowledge this is the first study that investigated the effect of ε4- *TOMM40* ‘523 haplotype variations on the risk of incident AD dementia among African Americans. Our data came from a diverse group of community based older persons who were free of AD at enrollment and followed longitudinally for incident events. The prospective nature of the design avoids potential bias due to prevalent AD. Uniform and structured decision rules for AD diagnosis were applied across all three studies. Limitations of this study are also noted. These are voluntary cohorts and participants are older and have higher education than the general population. The findings await replications from other studies.

## Supporting information

S1 File **Table A**. *APOE* ε4 and *TOMM40* ‘523-L with AD dementia in Caucasian Americans (matched sample) **Table B**. *APOE* ε4 and *TOMM40* ‘523-L with AD dementia in African Americans (matched sample).
(DOCX)

## References

[1] Tang MX, Stern Y, Marder K, Bell K, Gurland B, Lantigua R, et al The APOE-epsilon4 allele and the risk of Alzheimer disease among African Americans, whites, and Hispanics. Jama. 1998;279(10):751–5. Epub 1998/03/21. . 9508150 10.1001/jama.279.10.751

[2] Farrer LA, Cupples LA, Haines JL, Hyman B, Kukull WA, Mayeux R, et al Effects of age, sex, and ethnicity on the association between apolipoprotein E genotype and Alzheimer disease. A meta-analysis. APOE and Alzheimer Disease Meta Analysis Consortium. Jama. 1997;278(16):1349–56. Epub 1997/10/29. . 9343467

[3] Evans DA, Bennett DA, Wilson RS, Bienias JL, Morris MC, Scherr PA, et al Incidence of Alzheimer disease in a biracial urban community: relation to apolipoprotein E allele status. Archives of neurology. 2003;60(2):185–9. Epub 2003/02/13. . 12580702 10.1001/archneur.60.2.185

[4] Tycko B, Lee JH, Ciappa A, Saxena A, Li CM, Feng L, et al APOE and APOC1 promoter polymorphisms and the risk of Alzheimer disease in African American and Caribbean Hispanic individuals. Archives of neurology. 2004;61(9):1434–9. Epub 2004/09/15. doi: 10.1001/archneur.61.9.1434 . 15364690 10.1001/archneur.61.9.1434

[5] Roses AD, Lutz MW, Crenshaw DG, Grossman I, Saunders AM, Gottschalk WK. TOMM40 and APOE: Requirements for replication studies of association with age of disease onset and enrichment of a clinical trial. Alzheimer's & dementia: the journal of the Alzheimer's Association. 2013;9(2):132–6. Epub 2013/01/22. doi: 10.1016/j.jalz.2012.10.009 . 23333464 10.1016/j.jalz.2012.10.009

[6] Lutz MW, Crenshaw DG, Saunders AM, Roses AD. Genetic variation at a single locus and age of onset for Alzheimer's disease. Alzheimer's & dementia: the journal of the Alzheimer's Association. 2010;6(2):125–31. Epub 2010/03/20. doi: 10.1016/j.jalz.2010.01.011 ; 20298972 10.1016/j.jalz.2010.01.011 PMC2874876

[7] Cruchaga C, Nowotny P, Kauwe JS, Ridge PG, Mayo K, Bertelsen S, et al Association and expression analyses with single-nucleotide polymorphisms in TOMM40 in Alzheimer disease. Archives of neurology. 2011;68(8):1013–9. Epub 2011/08/10. doi: 10.1001/archneurol.2011.155 ; 21825236 10.1001/archneurol.2011.155 PMC3204798

[8] Yu L, Lutz MW, Wilson RS, Burns DK, Roses AD, Saunders AM, et al TOMM40'523 variant and cognitive decline in older persons with APOE epsilon3/3 genotype. Neurology. 2017;88(7):661–8. Epub 2017/01/22. doi: 10.1212/WNL.0000000000003614 ; 28108637 10.1212/WNL.0000000000003614 PMC5317377

[9] Roses AD, Lutz MW, Saunders AM, Goldgaber D, Saul R, Sundseth SS, et al African-American TOMM40'523-APOE haplotypes are admixture of West African and Caucasian alleles. Alzheimer's & dementia: the journal of the Alzheimer's Association. 2014;10(6):592–601 e2. Epub 2014/09/28. doi: 10.1016/j.jalz.2014.06.009 . 25260913 10.1016/j.jalz.2014.06.009

[10] Bennett DA, Schneider JA, Arvanitakis Z, Wilson RS. Overview and findings from the Religious Orders Study. Current Alzheimer research. 2012;9(6):628–45. ; 22471860 10.2174/156720512801322573 PMC3409291

[11] Bennett DA, Schneider JA, Buchman AS, Barnes LL, Boyle PA, Wilson RS. Overview and findings from the Rush Memory and Aging Project. Current Alzheimer research. 2012;9(6):646–63. ; 22471867 10.2174/156720512801322663 PMC3439198

[12] Barnes LL, Shah RC, Aggarwal NT, Bennett DA, Schneider JA. The Minority Aging Research Study: ongoing efforts to obtain brain donation in African Americans without dementia. Current Alzheimer research. 2012;9(6):734–45. Epub 2012/04/05. ; 22471868 10.2174/156720512801322627 PMC3409294

[13] McKhann G, Drachman D, Folstein M, Katzman R, Price D, Stadlan EM. Clinical diagnosis of Alzheimer's disease: report of the NINCDS-ADRDA Work Group under the auspices of Department of Health and Human Services Task Force on Alzheimer's Disease. Neurology. 1984;34(7):939–44. Epub 1984/07/01. . 6610841 10.1212/wnl.34.7.939

[14] Bennett DA, Schneider JA, Aggarwal NT, Arvanitakis Z, Shah RC, Kelly JF, et al Decision rules guiding the clinical diagnosis of Alzheimer's disease in two community-based cohort studies compared to standard practice in a clinic-based cohort study. Neuroepidemiology. 2006;27(3):169–76. Epub 2006/10/13. doi: 10.1159/000096129 . 17035694 10.1159/000096129

[15] Cohen J. A Coefficient of Agreement for Nominal Scales. Educational and Psychological Measurement. 1960;20(1):37–46.

[16] Chu SH, Roeder K, Ferrell RE, Devlin B, DeMichele-Sweet MA, Kamboh MI, et al TOMM40 poly-T repeat lengths, age of onset and psychosis risk in Alzheimer disease. Neurobiology of aging. 2011;32(12):2328 e1–9. Epub 2011/08/09. ; doi: 10.1016/j.neurobiolaging.2011.06.016 21820212 10.1016/j.neurobiolaging.2011.06.016 PMC3192304

[17] Crenshaw DG, Gottschalk WK, Lutz MW, Grossman I, Saunders AM, Burke JR, et al Using genetics to enable studies on the prevention of Alzheimer's disease. Clinical pharmacology and therapeutics. 2013;93(2):177–85. Epub 2012/12/20. doi: 10.1038/clpt.2012.222 ; 23249780 10.1038/clpt.2012.222 PMC4131283

[18] Jun G, Vardarajan BN, Buros J, Yu CE, Hawk MV, Dombroski BA, et al Comprehensive search for Alzheimer disease susceptibility loci in the APOE region. Archives of neurology. 2012;69(10):1270–9. Epub 2012/08/08. doi: 10.1001/archneurol.2012.2052 ; 22869155 10.1001/archneurol.2012.2052 PMC3579659

[19] Maestre G, Ottman R, Stern Y, Gurland B, Chun M, Tang MX, et al Apolipoprotein E and Alzheimer's disease: ethnic variation in genotypic risks. Annals of neurology. 1995;37(2):254–9. doi: 10.1002/ana.410370217 7847867 10.1002/ana.410370217

[20] Evans DA, Beckett LA, Field TS, Feng L, Albert MS, Bennett DA, et al Apolipoprotein E epsilon4 and incidence of Alzheimer disease in a community population of older persons. Jama. 1997;277(10):822–4. Epub 1997/03/12. . 9052713

[21] Slooter AJ, Cruts M, Hofman A, Koudstaal PJ, van der Kuip D, de Ridder MA, et al The impact of APOE on myocardial infarction, stroke, and dementia: the Rotterdam Study. Neurology. 2004;62(7):1196–8. Epub 2004/04/14. . 15079025 10.1212/01.wnl.0000118302.66674.e1

[22] Linnertz C, Saunders AM, Lutz MW, Crenshaw DM, Grossman I, Burns DK, et al Characterization of the poly-T variant in the TOMM40 gene in diverse populations. PloS one. 2012;7(2):e30994 Epub 2012/02/24. doi: 10.1371/journal.pone.0030994 ; 22359560 10.1371/journal.pone.0030994 PMC3281049

[23] Graff-Radford NR, Green RC, Go RC, Hutton ML, Edeki T, Bachman D, et al Association between apolipoprotein E genotype and Alzheimer disease in African American subjects. Archives of neurology. 2002;59(4):594–600. 11939894 10.1001/archneur.59.4.594

[24] Reitz C, Jun G, Naj A, Rajbhandary R, Vardarajan BN, Wang L-S, et al Variants in the ATP-binding cassette transporter (ABCA7), apolipoprotein E ϵ4, and the risk of late-onset Alzheimer disease in African Americans. Jama. 2013;309(14):1483–92. doi: 10.1001/jama.2013.2973 23571587 10.1001/jama.2013.2973 PMC3667653

[25] Logue MW, Schu M, Vardarajan BN, Buros J, Green RC, Go RC, et al A comprehensive genetic association study of Alzheimer disease in African Americans. Archives of neurology. 2011;68(12):1569–79. doi: 10.1001/archneurol.2011.646 22159054 10.1001/archneurol.2011.646 PMC3356921

[26] Barnes LL, Arvanitakis Z, Yu L, Kelly J, De Jager PL, Bennett DA. Apolipoprotein E and change in episodic memory in blacks and whites. Neuroepidemiology. 2013;40(3):211–9. Epub 2013/02/01. doi: 10.1159/000342778 ; 23364031 10.1159/000342778 PMC3645297

[27] Evans DA, Bennett DA, Wilson RS, Bienias JL, Morris MC, Scherr PA, et al Incidence of Alzheimer disease in a biracial urban community: relation to apolipoprotein E allele status. Archives of neurology. 2003;60(2):185–9. 12580702 10.1001/archneur.60.2.185

[28] Murrell JR, Price B, Lane KA, Baiyewu O, Gureje O, Ogunniyi A, et al Association of apolipoprotein E genotype and Alzheimer disease in African Americans. Archives of neurology. 2006;63(3):431–4. doi: 10.1001/archneur.63.3.431 16533971 10.1001/archneur.63.3.431 PMC3203415

[29] Hendrie HC, Ogunniyi A, Hall KS, Baiyewu O, Unverzagt FW, Gureje O, et al Incidence of dementia and Alzheimer disease in 2 communities: Yoruba residing in Ibadan, Nigeria, and African Americans residing in Indianapolis, Indiana. Jama. 2001;285(6):739–47. 11176911 10.1001/jama.285.6.739

[30] Gureje O, Ogunniyi A, Baiyewu O, Price B, Unverzagt FW, Evans RM, et al APOE epsilon4 is not associated with Alzheimer's disease in elderly Nigerians. Ann Neurol. 2006;59(1):182–5. Epub 2005/11/10. doi: 10.1002/ana.20694 ; 16278853 10.1002/ana.20694 PMC2855121

[31] Kline A. Apolipoprotein E, amyloid-ss clearance and therapeutic opportunities in Alzheimer's disease. Alzheimer's research & therapy. 2012;4(4):32 Epub 2012/08/30. doi: 10.1186/alzrt135 ; 22929359 10.1186/alzrt135 PMC3506946

[32] Verghese PB, Castellano JM, Holtzman DM. Apolipoprotein E in Alzheimer's disease and other neurological disorders. The Lancet Neurology. 2011;10(3):241–52. Epub 2011/02/26. doi: 10.1016/S1474-4422(10)70325-2 ; 21349439 10.1016/S1474-4422(10)70325-2 PMC3132088

[33] Bales KR, Liu F, Wu S, Lin S, Koger D, DeLong C, et al Human APOE isoform-dependent effects on brain beta-amyloid levels in PDAPP transgenic mice. The Journal of neuroscience: the official journal of the Society for Neuroscience. 2009;29(21):6771–9. Epub 2009/05/29. doi: 10.1523/jneurosci.0887-09.2009 . 19474305 10.1523/JNEUROSCI.0887-09.2009 PMC6665579

[34] Castellano JM, Kim J, Stewart FR, Jiang H, DeMattos RB, Patterson BW, et al Human apoE isoforms differentially regulate brain amyloid-beta peptide clearance. Science translational medicine. 2011;3(89):89ra57. Epub 2011/07/01. doi: 10.1126/scitranslmed.3002156 ; 21715678 10.1126/scitranslmed.3002156 PMC3192364

[35] Reiman EM, Chen K, Liu X, Bandy D, Yu M, Lee W, et al Fibrillar amyloid-beta burden in cognitively normal people at 3 levels of genetic risk for Alzheimer's disease. Proceedings of the National Academy of Sciences of the United States of America. 2009;106(16):6820–5. Epub 2009/04/07. doi: 10.1073/pnas.0900345106 ; 19346482 10.1073/pnas.0900345106 PMC2665196

[36] Drzezga A, Grimmer T, Henriksen G, Muhlau M, Perneczky R, Miederer I, et al Effect of APOE genotype on amyloid plaque load and gray matter volume in Alzheimer disease. Neurology. 2009;72(17):1487–94. Epub 2009/04/03. doi: 10.1212/WNL.0b013e3181a2e8d0 . 19339712 10.1212/WNL.0b013e3181a2e8d0

[37] Bennett DA, Schneider JA, Wilson RS, Bienias JL, Berry-Kravis E, Arnold SE. Amyloid mediates the association of apolipoprotein E e4 allele to cognitive function in older people. Journal of neurology, neurosurgery, and psychiatry. 2005;76(9):1194–9. Epub 2005/08/19. doi: 10.1136/jnnp.2004.054445 ; 16107349 10.1136/jnnp.2004.054445 PMC1739810

[38] Mortimer JA, Snowdon DA, Markesbery WR. The effect of APOE-epsilon4 on dementia is mediated by Alzheimer neuropathology. Alzheimer disease and associated disorders. 2009;23(2):152–7. Epub 2009/06/03. ; 19484916 10.1097/wad.0b013e318190a855 PMC2752689

[39] Chiba-Falek O, Lutz MW. Towards precision medicine in Alzheimer’s disease: deciphering genetic data to establish informative biomarkers. Expert Review of Precision Medicine and Drug Development. 2017:1–9. doi: 10.1080/23808993.2017.1286227 10.1080/23808993.2017.1286227 PMC5605187 28944295

[40] Akai J, Kimura A, Hata RI. Transcriptional regulation of the human type I collagen alpha2 (COL1A2) gene by the combination of two dinucleotide repeats. Gene. 1999;239(1):65–73. . 10571035 10.1016/s0378-1119(99)00380-7

[41] Chiba-Falek O, Nussbaum RL. Effect of allelic variation at the NACP-Rep1 repeat upstream of the alpha-synuclein gene (SNCA) on transcription in a cell culture luciferase reporter system. Hum Mol Genet. 2001;10(26):3101–9. Epub 2001/12/26. . 11751692 10.1093/hmg/10.26.3101

[42] Okladnova O, Syagailo YV, Tranitz M, Stober G, Riederer P, Mossner R, et al A promoter-associated polymorphic repeat modulates PAX-6 expression in human brain. Biochem Biophys Res Commun. 1998;248(2):402–5. doi: 10.1006/bbrc.1998.8972 . 9675149 10.1006/bbrc.1998.8972

[43] Peters DG, Kassam A, St Jean PL, Yonas H, Ferrell RE. Functional polymorphism in the matrix metalloproteinase-9 promoter as a potential risk factor for intracranial aneurysm. Stroke. 1999;30(12):2612–6. . 10582986 10.1161/01.str.30.12.2612

[44] Searle S, Blackwell JM. Evidence for a functional repeat polymorphism in the promoter of the human NRAMP1 gene that correlates with autoimmune versus infectious disease susceptibility. J Med Genet. 1999;36(4):295–9. . 10227396 PMC1734346

[45] Shimajiri S, Arima N, Tanimoto A, Murata Y, Hamada T, Wang KY, et al Shortened microsatellite d(CA)21 sequence down-regulates promoter activity of matrix metalloproteinase 9 gene. FEBS Lett. 1999;455(1–2):70–4. . 10428474 10.1016/s0014-5793(99)00863-7

[46] Hefferon TW, Groman JD, Yurk CE, Cutting GR. A variable dinucleotide repeat in the CFTR gene contributes to phenotype diversity by forming RNA secondary structures that alter splicing. Proceedings of the National Academy of Sciences of the United States of America. 2004;101(10):3504–9. doi: 10.1073/pnas.0400182101 ; 14993601 10.1073/pnas.0400182101 PMC373492

[47] Mirkin SM. Expandable DNA repeats and human disease. Nature. 2007;447(7147):932–40. doi: 10.1038/nature05977 . 17581576 10.1038/nature05977

[48] Pearson CE, Nichol Edamura K, Cleary JD. Repeat instability: mechanisms of dynamic mutations. Nature reviews Genetics. 2005;6(10):729–42. doi: 10.1038/nrg1689 . 16205713 10.1038/nrg1689

[49] Willems T, Gymrek M, Highnam G, Genomes Project C, Mittelman D, Erlich Y. The landscape of human STR variation. Genome Res. 2014;24(11):1894–904. doi: 10.1101/gr.177774.114 ; 25135957 10.1101/gr.177774.114 PMC4216929

[50] Linnertz C, Anderson L, Gottschalk W, Crenshaw D, Lutz MW, Allen J, et al The cis-regulatory effect of an Alzheimer's disease-associated poly-T locus on expression of TOMM40 and apolipoprotein E genes. Alzheimer's & dementia: the journal of the Alzheimer's Association. 2014;10(5):541–51. Epub 2014/01/21. doi: 10.1016/j.jalz.2013.08.280 ; 24439168 10.1016/j.jalz.2013.08.280 PMC4098029

[51] Payton A, Sindrewicz P, Pessoa V, Platt H, Horan M, Ollier W, et al A TOMM40 poly-T variant modulates gene expression and is associated with vocabulary ability and decline in nonpathologic aging. Neurobiology of aging. 2015 doi: 10.1016/j.neurobiolaging.2015.11.017 . 26742953 10.1016/j.neurobiolaging.2015.11.017

[52] Bekris LM, Lutz F, Yu C-E. Functional analysis of APOE locus genetic variation implicates regional enhancers in the regulation of both TOMM40 and APOE. J Hum Genet. 2012;57(1):18–25. doi: 10.1038/jhg.2011.123 22089642 10.1038/jhg.2011.123 PMC3266441

