# Supplementary material for: APOE ε4-TOMM40 ‘523 haplotypes and the risk of Alzheimer’s disease in older Caucasian and African Americans
Source: PLoS One. 2017 Jul 3;12(7):e0180356. doi: 10.1371/journal.pone.0180356 (PMC5495438; doi:10.1371/journal.pone.0180356)
Supplement: S1 File — Table A. APOE ε4 and TOMM40 ‘523-L with AD dementia in Caucasian Americans (matched sample) Table B. APOE ε4 and TOMM40 ‘523-L with AD dementia in African Americans (matched sample). (DOCX) [file pone.0180356.s001.docx]

**Table A *APOE* ε4 and *TOMM40* ‘523-L with AD dementia in Caucasian Americans (matched sample)***

|  | HR (95% CI, *p*) | HR (95% CI, *p*) |
| --- | --- | --- |
| Age | 1.12 (1.06-1.17, <0.001) | 1.12 (1.06-1.17, <0.001) |
| Male sex | 0.60 (0.25-1.44, 0.249) | 0.57 (0.24-1.37, 0.206) |
| Education | 0.96 (0.86-1.08, 0.517) | 0.97 (0.86-1.08, 0.552) |
| ε3/4 heterozygosity | 3.19 (1.64-6.20, <0.001) | - |
| ε4/4 homozygosity | 1.84 (0.24-14.11, 0.558) | - |
| ‘523-L heterozygosity | - | 3.26 (1.63-6.54, <0.001) |
| ‘523-L homozygosity | - | 1.76 (0.23-13.50, 0.587) |

HR: Hazard ratio; CI: Confidence interval

The result in column 2 was from a Cox proportional hazards model which examined *APOE* ε4 genotypes on incident AD dementia, and the result in column 3 was from a separate Cox model which examined *TOMM40* ‘523-L genotypes on incident AD dementia.

*These results were based on a subset of Caucasian Americans (N=464) that were 1-to-1 matched to African Americans by age, sex, education, length of follow-up as well as vital status.

**Table B *APOE* ε4 and *TOMM40* ‘523-L with AD dementia in African Americans (matched sample)***

|  | HR (95% CI, *p*) | HR (95% CI, *p*) |
| --- | --- | --- |
| Age | 1.13 (1.08-1.19, <0.001) | 1.12 (1.07-1.17, <0.001) |
| Male sex | 1.54 (0.82-2.88, 0.177) | 1.57 (0.84-2.92, 0.159) |
| Education | 1.09 (0.99-1.20, 0.084) | 1.09 (0.99-1.19, 0.079) |
| ε3/4 heterozygosity | 1.27 (0.65-2.48, 0.493) | - |
| ε4/4 homozygosity | 7.24 (3.10-16.93, <0.001) | - |
| ‘523-L heterozygosity | - | 2.14 (1.11-4.12, 0.024) |
| ‘523-L homozygosity | - | 1.71 (0.23-12.89, 0.605) |

HR: Hazard ratio; CI: Confidence interval

The result in column 2 was from a Cox proportional hazards model which examined *APOE* ε4 genotypes on incident AD dementia, and the result in column 3 was from a separate Cox model which examined *TOMM40* ‘523-L genotypes on incident AD dementia.

*These results were based on a subset of African Americans (N=464) that were 1-to-1 matched to Caucasian Americans by age, sex, education, length of follow-up as well as vital status.
